# Digitization of One-Piece Oral Implants: A Feasibility Study

**DOI:** 10.3390/ma13081990

**Published:** 2020-04-24

**Authors:** Stefano Pieralli, Benedikt Christopher Spies, Luisa Valentina Kohnen, Florian Beuer, Christian Wesemann

**Affiliations:** 1 Department of Prosthodontics, Geriatric Dentistry and Craniomandibular Disorders, Charité—Universitätsmedizin Berlin, corporate member of Freie Universität Berlin, Humboldt-Universität zu Berlin, and Berlin Institute of Health, Aßmannshauser Str. 4-6, 14197 Berlin, Germany; stefano.pieralli@uniklinik-freiburg.de (S.P.); luisa-valentina.kohnen@charite.de (L.V.K.); florian.beuer@charite.de (F.B.); christian.wesemann@charite.de (C.W.); 2 Department of Prosthetic Dentistry, Faculty of Medicine, Center for Dental Medicine, Medical Center—University of Freiburg, Hugstetter Str. 55, 79106 Freiburg, Germany

**Keywords:** accuracy, ceramics, dental implants, digital impression, intraoral scanner, one-piece dental implant, trueness

## Abstract

For digital impression-making of two-piece oral implants, scan bodies are used to transfer the exact intraoral implant position to the dental laboratory. In this in vitro investigation, the accuracy of digitizing a one-piece ceramic oral implant without a scan body (OC) was compared to that of a standard two-piece titanium implant with a scan body (TT) and a preparation of a natural single tooth (ST). Furthermore, incomplete scans of OC simulating clinical compromising situations (OC_1–4_) were redesigned using a virtual reconstruction tool (RT) and superimposed to OC. OC and TT oral implants and one ST were inserted into a mandible typodont model and digitized (*N* = 13) using two different intraoral scanners. The resulting virtual datasets were superimposed onto a three-dimensional (3D) laser scanner-based reference. Test and reference groups were aligned using an inspection software according to a best-fit algorithm, and circumferential as well as marginal discrepancies were measured. For the statistical evaluation, multivariate analyses of variance with post-hoc Tukey tests and students *t*-tests to compare both scanners were performed. A total of 182 datasets were analyzed. For circumferential deviations, no significant differences were found between ST, TT, and OC (*p* > 0.964), but increased deviations for OC_1–4_ (*p* < 0.001) were observed. The measurements of the marginal deviations revealed that ST had the smallest deviations, and that there were no significant differences between TT, OC, and OC_1–4_ (*p* > 0.979). Except for marginal deviation of OC (*p* < 0.001), the outcome was not affected by the scanner. Within the limitations of this study, digitization of OC is as accurate as that of TT, but less than that of ST. In the case of known geometries, post-processing of compromised scans with a virtual reconstruction results in accurate data.

## 1. Introduction

For the manufacturing of a tooth- or implant-borne prosthesis, the shape and position of teeth and oral implants need to be transferred to the dental laboratory in a highly accurate and reliable procedure. In terms of conventional impression-making techniques, there are significant differences between teeth and oral implants [1,2]. Natural teeth present an individual shape, margin preparation line, and emergence profile. To obtain an accurate impression of subgingival areas, methods to displace the gingiva, such as retraction cords, are needed [3]. These procedures are both time-consuming for the clinicians and uncomfortable for the patients [4,5]. In contrast, the implant-abutment connection (IAC) of an oral implant has a standardized geometry, and in-lab implant replicas with identical IACs are used for subsequent manufacturing of the restorations [6]. To transfer the precise intraoral position of an implant in a conventional workflow, an impression coping of identical IAC is screwed into the implant. For the “transfer method” impression technique a transfer cap attached to the impression coping remains in the impression material after setting and removal from the oral cavity of the closed tray [7]. This allows repositioning of the impression coping with an attached lab-replica of the implant prior to pouring. For the “pick up method,” the abutment screw of the impression coping needs to be unscrewed from the fixture after setting of the impression material, allowing the coping to remain “fixed” within it [8]. In contrast to the “transfer method,” no transfer cap, but instead an open impression tray is necessary. Furthermore, a screwless approach using impression components to snap onto the transgingival part of the implant is available, but scarcely reported in the literature [9,10]. As passive fit of implant-borne superstructures seems mandatory for long-term clinical success [11,12,13], it is recommended to splint impression copings in the case of multiple adjacent implants to minimize the risk of discrepancies caused by deformation of the impression material [11]. The majority of in vitro investigations have confirmed the benefit of splinted copings in edentulous and partially edentulous patients in the case of ≥4 implants [12]. 

Digitization of oral implants is based on the acquisition of images to transfer the implant position to a virtual model [13], thereby avoiding the production and storage of stone casts [14]. During impression-making, this procedure allows for simultaneous visualization of the area of interest [14], and delivers a more convenient treatment for patients compared to conventional impressions [15]. According to a recent Delphi study, the intraoral digitization of implants, followed by computer-aided design and manufacturing (computer-aided design (CAD)/computer-aided manufacturing (CAM)) of the implant-supported restorations in a fully digital workflow, is expected to replace conventional techniques within the next decade [16]. To digitize standard two-piece implants, a scan body used as reference geometry is screwed into the implant (“virtual impression coping”) and its position is recorded by means of an intraoral scanner (IOS) [17]. A recent in vitro investigation demonstrated the influence of both the scan strategy and the geometry of the scan bodies on the accuracy of the digitization process [18]. Irrespective of the system, the fit of the final restoration depends on the accuracy of the previous intraoral scan. 

Besides two-piece implants, one-piece fixtures are likewise available on the market and, among other materials, are made from zirconium dioxide (zirconia, ZrO_2_) [19]. These implants present a monobloc design, including the endosseous part and the abutment in one piece. In this case, prosthetic-oriented implant positioning is mandatory to prevent subsequent intraoral adjustments of the abutment portion due to prosthetic reasons (e.g., path of insertion), which are liable to weaken the entire implant–restoration complex [20]. Nevertheless, anatomical limitations such as a scarce vertical dimension of the alveolar bone may require a reduction of the abutment in both the vertical and horizontal directions, especially in the esthetic zone due to diverging angulation of the bone and anterior reconstructions [21]. Comparable to natural teeth, retraction cords might be needed to expose the implant shoulder for impression taking [22], whereas the presence of blood or saliva, as well as deeply inserted implants, can complicate the process.

When digitizing a one-piece implant, the implant abutment itself might serve as a reference geometry for reconstruction, thus avoiding the need for additional scan bodies. Furthermore, in the case of incomplete scans, a virtual reconstruction of missing parts based on the known geometry of the standardized abutment might still be possible. Therefore, this in vitro study focused on two aspects: (1) the accuracy of digital impression-making with unrestricted exposure of the abutment of a one-piece ceramic (OC) implant made from ZrO_2_, and (2) the feasibility to virtually reconstruct missing parts of a partial digitized abutment in case of a clinically compromised situation. A single-tooth (ST) preparation and a two-piece titanium (TT) implant with a scan body served as references for comparison. The null hypothesis assumed no difference in terms of trueness between digital impressions of ST, TT, OC, and virtually reconstructed scans of OC.

## 2. Materials and Methods

### 2.1. Reference Model

A reference typodont model (ANA-4, Frasaco, Tettnang, Germany) of the mandible was used. An OC revealing an endosseous length of 10 mm and width of 4 mm, a transgingival part 2 mm in height, and a 4° conical abutment 4.5 mm in height (ceramic.implant, vitaclinical, VITA Zahnfabrik, Bad Säckingen, Germany) was positioned in the fourth quadrant in the region of the second premolar. A TT (3.8 × 9 mm) (CAMLOG SCREW-LINE, Camlog, Basel, Switzerland) was installed on the contralateral side. A human first molar was prepared and placed in the fourth quadrant. The ST presented a rounded shoulder-shaped preparation margin with 1 mm circumferential and 1.5 mm occlusal substance removal. To obtain a virtual reference dataset, the model was digitized using a blue light three-dimensional (3D) LED scanner (D2000, 3shape, Copenhagen, Denmark), which shows a precision of 5 μm/8 μm (ISO 12836/implant).

### 2.2. Acquisition of the Test Datasets

Two different intraoral scanners were used to perform digital impressions: TRIOS 3, software version 1.4.7.3 (3shape) and Omnicam, software version 5.0.x (Dentsply–Sirona, York, Pennsylvania, USA). A standardized scan pattern was followed for all the scanning procedures and included both the examination area and the adjacent teeth. It started with a 45° angle from the vestibular aspect of the mesial adjacent tooth, switched to the lingual aspect until the distal end of the opposite adjacent tooth, switched to the buccal aspect, and returned to the starting point. All scans were performed solely by one trained operator (L.V.K.) under ceiling lighting. 

**Figure 1:**
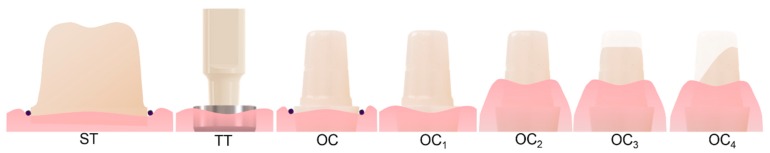
Schematic illustration of the investigation groups. ST, single-tooth preparation; TT, one-piece titanium implant with a scan body; OC, one-piece ZrO_2_ implant; OC_1_, OC for matching with reconstruction support; OC_2_, simulated subgingival insertion of 1.5 mm; OC_3_, additional occlusal reduction of 1 mm; OC_4_, additional lateral reduction of 45°.

For each of the examined samples (OC, TT, ST), 13 digital impressions with both scanners were taken, resulting in 78 datasets. The 26 scan files related to OC were subsequently duplicated and virtually modified (Figure 1). First (OC_1_), the scan of OC was superimposed with the original dataset of the implant abutment provided by the manufacturer and compared to the reference dataset. Second (OC_2_), a subgingival positioning of the implant shoulder was simulated. In this case, the lower third of the scanned implant head, which corresponds to 1.5 mm, was removed from all datasets. Third (OC_3_), an occlusal modification of the abutment geometry by grinding was simulated. In this case, the marginal reduction was followed by an occlusal reduction of the abutment of 1 mm, and the resulting datasets were stored. Fourth (OC_4_), a further lateral individualization of the implant abutment, corresponding to a lateral reduction of 45° to the vertical, followed the modifications adopted for OC_3_.

**Figure 2:**
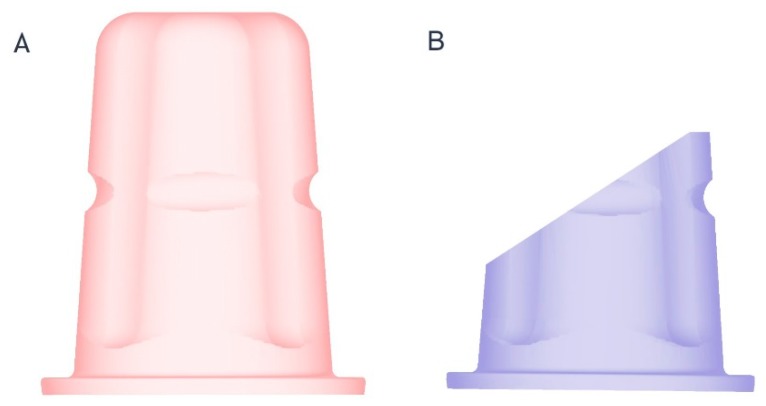
(**A**) Reconstruction tool for unmodified implants (OC_1,2_); (**B**) reconstruction tool for intraorally adjusted (OC_3,4_) implants.

The original implant geometry of OC, provided by the manufacturer in the form of a digital dataset (CAD File, ceramic.implant), was used to develop a virtual reconstruction tool (RT) (Figure 2). 

**Figure 3:**
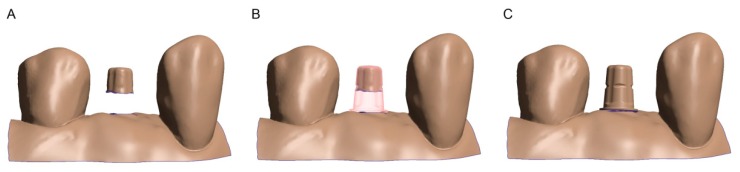
Reconstruction procedure: (**A**) OC_2_ with 1.5 mm missing data marginally; (**B**) OC_2_ matched with RT; (**C**) both datasets combined.

The developed RT was matched with the OC_1–4_ scans to reconstruct the missing areas of the digitized abutment. For this purpose, both datasets were aligned using an inspection software (Geomagic Control X, 3-D-Systems, Rock Hill, South Carolina, USA) via a 3-point alignment, and then matched following a best-fit superimposition algorithm, according to Gauss. Both datasets were merged using Boolean operators, with the final surface of the implant head consisting of the RT. In the second subgroup (OC_2_), the RT was merged into the marginal reduced scan files. In the third subgroup (OC_3_), the RT was matched to the marginal and occlusal reduced datasets based on the remaining implant areas. In the fourth subgroup (OC_4_), the remaining 30% of the implant surface after marginal, occlusal, and lateral reduction was used to match the RT and thus to recreate the missing data (Figure 3). The resulting subgroups from OC consisted of a further 104 modified and reconstructed datasets.

### 2.3. Data Analysis

To calculate the deviations of the test datasets, these were superimposed with the reference dataset using the Geomagic Control X software and a local best-fit algorithm. The superimposition was performed over the two respective adjacent teeth. Subsequently, 3D surface comparisons were performed, separately, for the circumferential and marginal areas of the datasets. Marginal areas were defined as the horizontal areas of the implant shoulder for OC, OC_1–4_, and TT, as well as the rounded shoulder preparation margin to the transition from round to conical areas for ST. The absolute values of the surface deviation were used for further evaluation of the deviations. Therefore, the definition of marginal deviation adopted in this article must be referred to a surface not a line. 

### 2.4. Statistical Analysis

Surface deviations of the study groups were separately analyzed for both scanners using multivariate analyses of variance (MANOVA) with post-hoc Tukey pairwise comparisons for the two dependent variables—circumferential and marginal deviations. In addition, both scanners were compared with students *t*-tests for the respective correlating groups. The analysis was performed with SPSS 22.0. The level of significance was set as *p* < 0.05. A priori sample size calculation based on five pilot scans of ST and OC revealed a required sample size of *n* = 13 to show differences with an α = 0.05 and a given Power of β = 0.80 as significant (Effect size *d* = 1.16)

## 3. Results

**Figure 4:**
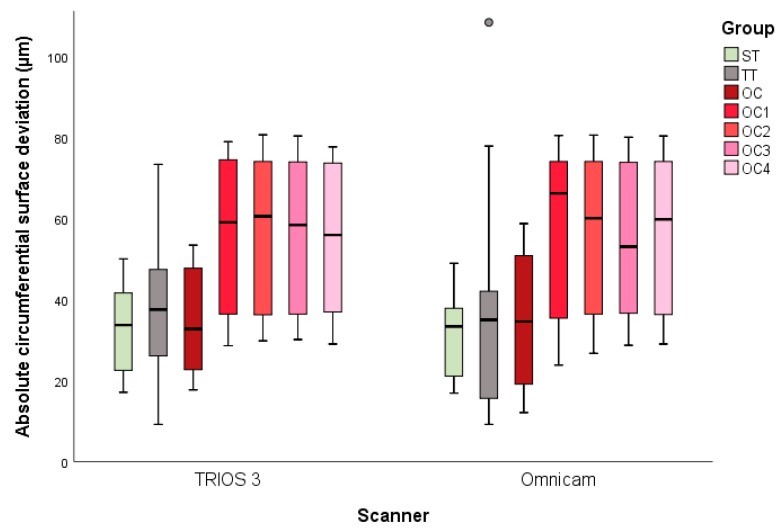
Box and whisker plots of the minimum, maximum, interquartile range, median, and outliers for the absolute circumferential surface deviations.

**Figure 5:**
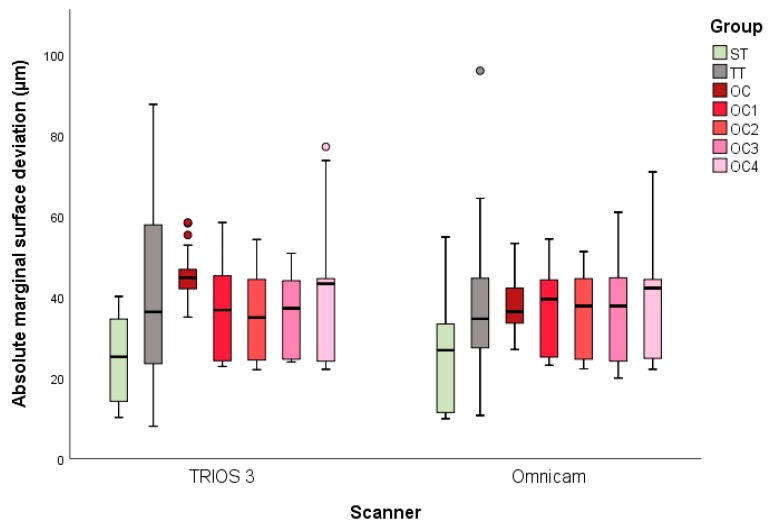
Box and whisker plots of the minimum, maximum, interquartile range, median, and outliers for the absolute marginal surface deviations.

For each of the seven groups (OC, OC_1–4_, ST, and TT), 13 digital impressions with two scanners each (TRIOS 3 and Omnicam) were performed, resulting in 182 datasets in total. The descriptive statistics of the absolute circumferential and marginal deviations are presented in Table 1. Figure 4 and Figure 5 present the results for the lateral and marginal deviations visualized as box and whisker plots. MANOVA revealed significant differences between the study groups for both circumferential *(p* < 0.001) and marginal deviations *(p* < 0.001).

### 3.1. Circumferential Deviation

The digital impressions obtained with TRIOS 3 showed a minor mean surface deviation when digitizing ST (33 ± 10 µm) (Figure 4). This outcome did not significantly differ from the digitization of TT (37 ± 17 µm; *p* = 0.964) or OC (35 ± 13 µm; *p* = 0.999) when using the same scanning system. The virtually reconstructed implants (OC_1–4_) showed significantly higher deviations than OC *(p* < 0.001), but no significant differences between the reconstructed groups were found *(p* > 0.999). Digitization with Omnicam likewise demonstrated no significant differences between ST, TT, and OC (*p* < 0.975). The lowest mean deviation value was recorded for ST (32 ± 11 µm), followed by OC (35 ± 16 µm) and then TT (35 ± 23 µm). The reconstructed implant scans showed higher mean deviation values than the aforementioned groups and ranged from OC_3_ (55 ± 19 µm) to OC_1_ (56 ± 21 µm). Finally, significant differences were found between OC and OC_1–4_ *(p* > 0.001), with no significant differences between the reconstructed groups (*p* > 0.999). The *t*-tests between the corresponding groups scanned with TRIOS 3 and Omnicam showed no significant differences (*p* > 0.459) 

### 3.2. Marginal Deviation

The digital impressions using TRIOS 3 showed that, of the tested groups, ST had the smallest deviation from the reference, with 24 ± 10 µm (Figure 5). These scans were significantly more accurate than for TT (40 ± 20 µm; *p* < 0.001) and OC (45 ± 6 µm; *p* < 0.001). Furthermore, compared to OC, all of the reconstructed groups (OC_1–4_) showed decreased mean marginal deviation values, between 35 ± 10 µm (OC_3_) and 38 ± 15 µm (OC_4_), and no significant differences between the groups were found, independent of the extent of the reconstruction (*p* > 0.979). The digitized data obtained with Omnicam showed similar results compared to TRIOS 3. In fact, significantly lower deviations were found for ST (26 ± 12 µm) compared to TT (41 ± 24 µm; *p* < 0.001) and OC (38 ± 6 µm; P < 0.007). In addition, the reconstructed implants (OC_1–4_) showed mean marginal deviations ranging between a minimum of 36 ± 10 µm (OC_1_) and a maximum of 37 ± 12 µm (OC_4_). No statistically significant differences between OC_1–4_ were found (*p* < 0.999). Finally, scan accuracy was not affected by the IOS used, except for OC *(p* < 0.001) which showed a lower deviation with Omnicam.

## 4. Discussion

The term accuracy is defined as a “closeness of agreement between a measured quantity value and a true quantity value of a measurand” [23] and refers to both the trueness and the precision of a system [24]. Trueness is related to the closest representation of the measurement and precision to the repeatability and consistency of the measurement itself. In this investigation, to the authors knowledge the first one evaluating digitization of OC, accuracy was assessed by measuring the deviation between the test groups (OC, ST, TT) and their superimposed reference using a surface matching software [25]. In particular, trueness was considered the mean deviation between the positive and negative values, and precision depended on the corresponding standard deviations, according to comparable studies [26,27]. Two different IOS systems were used for the digitization of OC, ST, and TT. A 3D laser scanner, to date considered a sophisticated instrument in terms of accuracy, delivered a reliable reference dataset. As reported by Flügge et al., the assessment of a true reference model represents a major issue, especially in vivo, when evaluating spatial deviations [8]. The in vitro design of this study allowed further favorable conditions: a simplified scanning procedure avoiding limitations in space and the absence of intraoral fluids. This might have led to more precise results compared to a clinical routine [28].

The primary aim of this investigation was to evaluate the feasibility of scan body-free digital impressions of OC compared to scans of TT with a scan body and ST. Statistical analysis showed that circumferential trueness of the test group (OC) was not significantly different from the control groups (TT and ST). In contrast, the marginal trueness of OC showed no statistically significant deviations compared to TT, but significant differences compared to ST. Therefore, the null hypothesis must be partially rejected, as ST was more accurate than OC analysis.

A secondary objective of this investigation was to evaluate the feasibility and accuracy of the virtual reconstruction of the abutment of OC based on simulated clinically compromised situations. For this purpose, a virtual RT was created based on the original virtual dataset of the abutment. The absence of significant differences between the reconstructed implants (OC_1–4_), irrespective of the extent of the reconstruction, indicates the feasibility of the virtual RT used in the present study and makes it recommendable for clinical application. However, when comparing the trueness of OC_1–4_ to OC, disparities between the two methods were found: circumferential deviations with reconstruction increased in OC_1–4_ compared to OC, while marginal deviations decreased. The highest mean deviation for OC_1–4_ for the circumferential aspect was 56 ± 21 µm (OC_1_-Omnicam) compared to 38 ± 15 µm (OC_4_-TRIOS 3) for the marginal aspect. To date, a clear definition of passive fit in implant prosthodontics is still not available in the literature [29], and marginal discrepancies in a range of 30–150 µm between implant and prosthetic components are considered acceptable [30]. Therefore, the results of this study are in accordance with the current literature.

**Figure 6:**
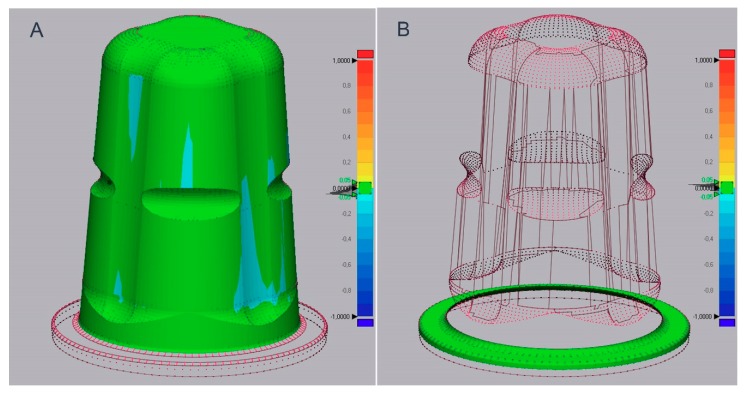
Representative measurements of OC_2_ with a tolerance of ±50 µm (green regions) for (**A**) circumferential deviations and (**B**) marginal deviations.

Virtual reconstruction of partial/compromised scans by means of digital post-processing with a given dataset simplified the simulated intraoral digitization process, showing that even one third of a captured abutment seems to be sufficient for reconstructive purposes. Another advantage in terms of standardization and automatization of the workflow is represented by the conversion of surface data from a polygonal free form into a standard geometry. This allows the automatized determination of the implant shoulder margin (Figure 6) and the possibility to predefine individualized spacer settings for subsequent cementation of the suprastructure.

The digital workflow, which is “expected to replace the traditional indirect methods used in traditional dentistry” [16], can be divided into three parts: computer-aided impression (CAI) of oral structures, and CAD and manufacturing (CAM) of the restoration [31]. Every step is associated with potential discrepancies that are propagated to the next stage. The evaluated spatial deviations of the digitized OC, ST, and TT assessed the accuracy of the first part of the digital workflow. In addition, reconstructed OC_1–4_ showed that a partial scan can be accurately reconstructed virtually during the second part of the process. The possibility to virtually reconstruct OC might be useful for the clinician, in particular in compromising clinical circumstances, and may permit a more patient-friendly impression-making procedure. Furthermore, CAD based on standard geometries instead of free-form surfaces would facilitate the subsequent workflow in the lab. To allow these procedures, the geometries of one-piece implants should be included in the databases of CAD programs. Therefore, the prosthetic disadvantage of one-piece implants would be partially compensated by avoiding the need for scan bodies; however, more investigations are necessary to confirm these findings in vivo. Comparative clinical studies might assess the reliability of the presented workflow also for similar implant systems. 

## 5. Conclusions

Taking the limitations of the present in vitro setup into account, digitization of one-piece zirconia implants without a scan body results in an accurate virtual model and presents a potential alternative to conventional impression-making methods. Incomplete scans can be digitally reconstructed based on a known abutment geometry.

## Figures and Tables

**Table 1:** Mean, standard deviation (SD), and confidence intervals (CIs) of the circumferential and marginal surface deviations. For each group n = 13.

IOS	Group	Circumferential Surface Deviations (µm)		Marginal Surface Deviations (µm)	
		Mean ± SD	95% CI	Mean ± SD	95% CI
**TRIOS 3**	ST	33 ± 10 ^a^	[28, 37]	24 ± 10 ^a^	[20, 28]
	TT	37 ± 17 ^a^	[30, 44]	40 ± 20 ^b^	[32, 49]
	OC	35 ± 13 ^a^	[30, 40]	45 ± 6 ^b^	[43, 48]
	OC_1_	55 ± 20 ^b^	[47, 63]	36 ± 11 ^b^	[31, 40]
	OC_2_	56 ± 20 ^b^	[48, 64]	36 ± 11 ^b^	[31, 40]
	OC_3_	55 ± 19 ^b^	[48, 63]	35 ± 10 ^b^	[31, 39]
	OC_4_	55 ± 19 ^b^	[47, 63]	38 ± 15 ^b^	[32, 44]
	Significance *	*F(6,175) = 10.66, p < 0.001*		*F(6,175) = 6.7, p < 0.001*	
**Omnicam**	ST	32 ± 11 ^a^	[28, 36]	26 ± 12 ^a^	[21, 30]
	TT	35 ± 23 ^a^	[23, 44]	41 ± 24 ^b^	[31, 50]
	OC	35 ± 16 ^a^	[16, 42]	38 ± 6 ^b^	[35, 41]
	OC_1_	56 ± 21 ^b^	[21, 64]	36 ± 10 ^b^	[32, 40]
	OC_2_	56 ± 20 ^b^	[20, 64]	36 ± 11 ^b^	[32, 40]
	OC_3_	55 ± 19 ^b^	[19, 63]	36 ± 12 ^b^	[31, 40]
	OC_4_	56 ± 20 ^b^	[20, 64]	37 ± 12 ^b^	[32, 42]
	Significance *	*F(6,175) = 10.62, p < 0.001*		*F(6,175) = 3.76, p < 0.001*	

Results labeled with the same superscript letter within a column were not significantly different (* one-way ANOVA with post-hoc Tukey test). IOS, intraoral scanner.
